# Supplementary figures and images for: Spherical harmonics texture extraction for versatile analysis of biological objects
Source: PLoS Comput Biol. 2025 Jan 29;21(1):e1012349. doi: 10.1371/journal.pcbi.1012349 (PMC11798461; doi:10.1371/journal.pcbi.1012349)

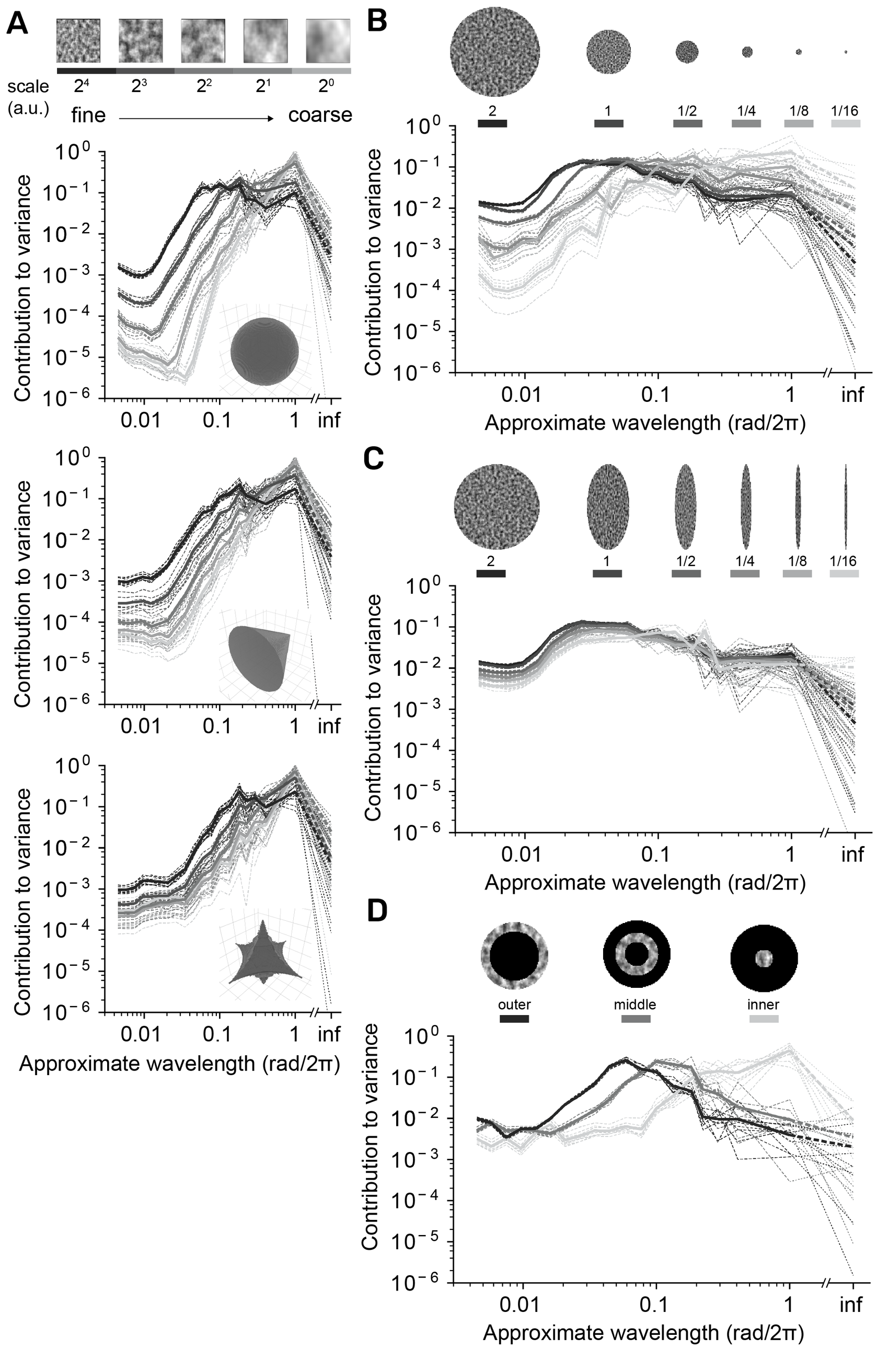

Supplement: S1 Fig — A) Spherical Texture quantification of differently shaped objects, filled with identical 3D test patterns at different scales, shows that the Spherical Texture quantification is internally consistent within the same shapes and comparable to other similar shapes. Insets: Mask shapes are shown as 3D surface renders. B) Spherical Texture quantifications of objects scaled to different sizes reflect the changes in the 3D pattern scale. The scales are given relative to the 80-pixel scaling used in the Spherical Texture quantification. C) The Spherical Texture quantification is robust against rescaling a single axis of objects to simulate anisotropic sampling and only shows a small loss in quality. D) Synthetic patterns clipped to different distances from the center of the object show that a pattern in the Spherical Texture will appear coarser if it is located closer to the center of the object. (TIF) [file pcbi.1012349.s001.tif]

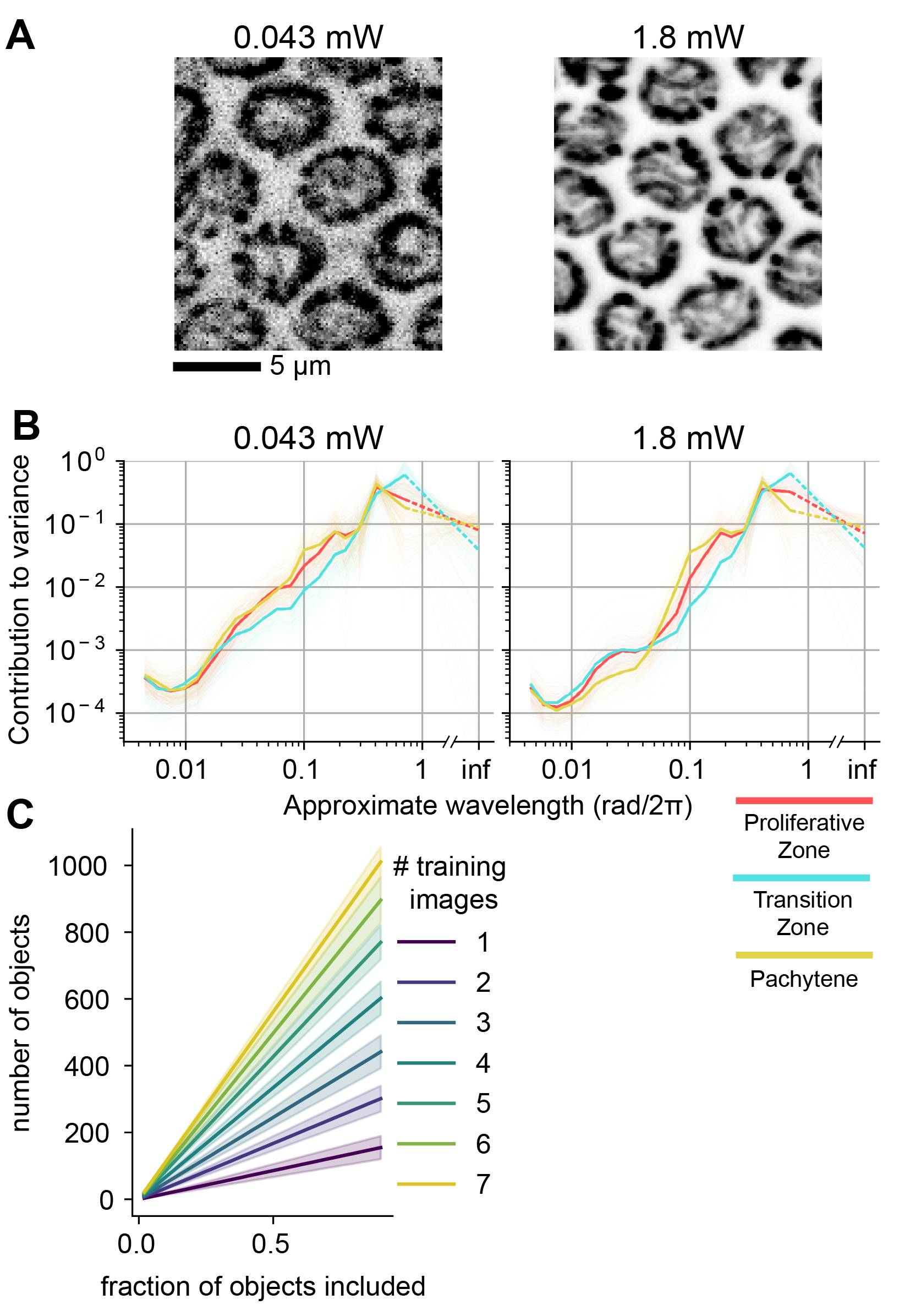

Supplement: S2 Fig — A) Two wild-type C. elegans datasets with different noise levels, acquired using different laser power (wattage delivered into the objective is given). B) Spherical Texture quantification of manually classified C. elegans nuclei in the two datasets acquired with different laser power. Here we see that large-scale (high wavelength) features remain, while smaller details (low wavelength) are noise-dependent. C) The average number of nuclei included for subsetting the C. elegans germline nucleus training dataset depicted in Fig 4C. (TIF) [file pcbi.1012349.s002.tif]

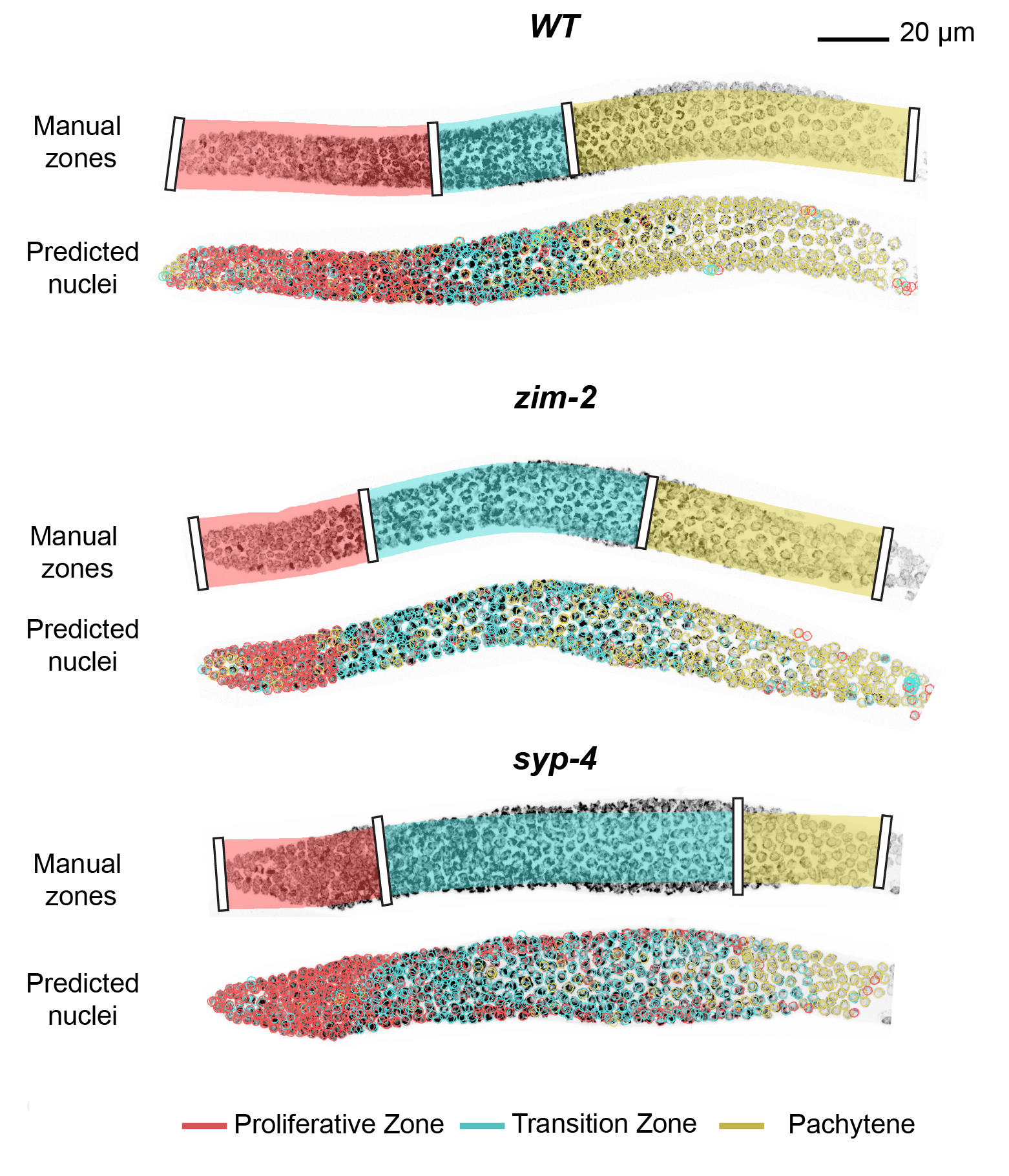

Supplement: S3 Fig — (TIF) [file pcbi.1012349.s003.tif]
